# Situational Analysis of Household Energy and Biomass Use and Associated Health Burden of Indoor Air Pollution and Mitigation Efforts in Pakistan

**DOI:** 10.3390/ijerph7072940

**Published:** 2010-06-20

**Authors:** Zafar Fatmi, Asma Rahman, Ambreen Kazi, M. Masood Kadir, Nalini Sathiakumar

**Affiliations:** 1 Division of Environmental Health Sciences, Department of Community Health Sciences, Aga Khan University, Stadium Road, P.O. Box 3500, Karachi, Pakistan; 2 Department of Community Health Sciences, Aga Khan University, Stadium Road, P.O. Box 3500, Karachi, Pakistan; E-Mails: asma.rahman@aku.edu (A.R); ambreen.kazi@aku.edu (A.K); masood.kadir@aku.edu (M.K); 3 Department of Epidemiology, School of Public Health, University of Alabama at Birmingham, RPHB 517D, 1665 University Blvd. Birmingham, AL 35294-0022, USA; E-Mail: NSathiakumar@ms.soph.uab.edu

**Keywords:** Indoor air pollution (IAP), Pakistan, health impact of IAP, control efforts for IAP

## Abstract

Biomass fuel burning leads to high levels of suspended particulate matter and hazardous chemicals in the indoor environment in countries where it is in common use, contributing significantly to indoor air pollution (IAP). A situational analysis of household energy and biomass use and associated health effects of IAP was conducted by reviewing published and un-published literature about the situation in Pakistan. In addition to attempt to quantify the burden of ill health due to IAP, this paper also appraises the mitigation measures undertaken to avert the problem in Pakistan. Unfortunately, IAP is still not a recognized environmental hazard in Pakistan and there are no policies and standards to control it at the household level. Only a few original studies related to health effects of IAP have been conducted, mainly on women’s health and birth outcome, and only a few governmental, non-governmental and academic institutions are working to improve the IAP situation by introducing improved stoves and renewable energy technology at a small scale. Control of IAP health hazards in Pakistan requires an initial meeting of the stakeholders to define a policy and an action agenda. Simultaneously, studies gathering evidence of impact of intervention through available technologies such as improved stoves would have favorable impact on the health, especially of women and children in Pakistan.

## 1. Introduction

Indoor air pollution (IAP), in general, is a major concern for both developed and developing countries. However, the gravity of the situation is far greater for the latter, particularly due to high reliance on solid fuels whose use are a major source of IAP in developing countries. Biomass refers to any plant or animal based material burned by humans, mainly for cooking, lighting and heating in homes. It includes wood, charcoal, agricultural residues and dung. It is estimated that globally more than three billion people currently rely on solid fuels, of which 2.4 billion use biomass fuels while the rest are dependent on coal, which is mostly being used in China. There are marked regional variations in biomass use, being as low as 20% in Europe and Central Asia and as high as 80% or more in Sub-Saharan Africa and South Asia [1]. Access to modern energy sources is a necessary requirement for economic and social development [2]. A large portion of the world’s population still does not have access to modern energy sources, mainly due to low income and the lack of availability of alternative, modern fuels. In the near term, it is not foreseeable that alternate cleaner fuels will become available in underserved areas.

In addition solid fuels are responsible for causing several adverse health effects on the population. Biomass burns incompletely, thus releasing, in addition to carbon dioxide, a multitude of complex chemicals including suspended particulate matter (SPM), carbon monoxide, formaldehyde, nitrogen dioxide, ozone and polycyclic aromatic hydrocarbons (PAH), among others. They are mostly burned in open fires or in three-stone stoves, leading to release of high levels of noxious chemicals. Exposure to these substances leads to increased risk of a variety of diseases including pneumonia, chronic respiratory diseases and lung cancer [3].

In simple terms, the effect on health due to exposure to biomass smoke can be determined by the duration of exposure along with the air concentration of each pollutant (pollution level*time). It can be measured in units of person-hour of exposure and measured directly through personal monitoring or indirectly through information on *pollutant concentration* and *activity patterns* [4]. Direct measures are more reliable than indirect measurement, but costly, so many estimates around the World are based on indirect measures.

The amount of fuel burnt individually at the household level may be much less than the amount in use in industries, and may therefore contribute less to ambient air pollution; however, its impact on health is greater due to its presence in the indoor environment and the greater amount of time spent indoors by humans. Of the two million estimated deaths due to air pollution 1.2 million are attributed to IAP [5]. Moreover, women and young children of developing countries are at greatest risk because of their gender roles and household responsibilities and behaviors – cooking and spending lots of time indoors and keeping children with them while cooking – resulting in high exposure to IAP. In addition, children are particularly vulnerable to IAP because their metabolic pathways are underdeveloped and immature and they are not able to completely get rid of the IAP compounds from their body.

Pakistan has a population of 170 million and ranks as the 6th most populous country in the World. It also exhibits the highest growth rate in the South Asian region [6]. The average household size in Pakistan is estimated to be 6.8 persons, its gross national product (GNP) is $689 (2004) and 2/3rds of the population is rural [7]. Forty nine percent of the population is estimated to belong to the low socioeconomic group, mostly in the rural areas [8]. Mortality figures in Pakistan for infants, children under 5-years and for mothers are dismal. The number of deaths in Pakistan attributed to ARI among children under 5 years, according to WHO data, has been estimated to be 51,760 [9].

National estimates for health burden of IAP have vital importance for a developing country like Pakistan where the majority of the population use biomass fuel as the main source of energy. This may be considered as a first step towards policy development. The objective of this review was to perform a situational analysis regarding household energy use and the health burden of IAP in Pakistan. Furthermore, the paper focuses on the adverse health effects of IAP on women and children in Pakistan. A review on the capacity of institutions and organizations involved in mitigation of IAP has also been done, and recommendations made for improvement of the IAP situation in Pakistan.

## 2. Methodology

A review of literature was done through published and un-published sources regarding IAP in Pakistan. In the first attempt PubMed sources were searched using MeSH words “indoor air pollution AND Pakistan” and “indoor air pollution AND health AND Pakistan”. A total of 19 published articles were retrieved, of which nine were directly related to biomass use and its health effects and seven were related to the health effects of radon. The remaining three were not specifically focused on IAP, but indoor air was considered as one of the risk factors for disease development. The term “household energy” and “Pakistan” retrieved no relevant results in PubMed. Although Google may not necessarily be considered a suitable search engine for conducting scientific research; however, to supplement our search we expanded it to include this search tool. A significant number of literature references related to agriculture and use of biomass as household energy, its implication for deforestation and ecological environment was found. None of this literature discussed the health effects of IAP, however it provided detailed information regarding household energy and biomass use in Pakistan. Some literature also provided information on the gender roles of women for household energy consumption. Therefore, the literature related to household energy use was cited although its detailed environmental consequences and its trend were not included in our review.

Furthermore, we accessed information from governmental and non-governmental agencies (NGOs) working on environmental health in Pakistan. The Pakistan Environmental Protection Agency (EPA) and The World Conversation Union (IUCN) offices were contacted to identify agencies and institutions working on the environment and IAP in Pakistan. A list of agencies and organizations working in these fields that had undertaken or have the potential to undertake work on IAP and their efforts have also been discussed. This grey literature was mostly concerned with ecological health of flora and fauna and did not provide information on human health related to IAP.

Published and unpublished sources were used to provide summaries on household energy and biomass use and adverse health effects of IAP. Where information was found to be insufficient extrapolation has been done to provide possible estimates from small scale studies. A review of information regarding the mitigation measures and the projects undertaken by the government and non-government organizations were also summarized to determine the gaps, their potential for upscaling, and the lessons learned.

## 3. Results and Discussion

### 3.1. Household energy use and estimated indoor air pollution in Pakistan

Although energy consumption per capita in Pakistan has tripled since 1970, it is still among the low carbon emission countries. Carbon emission and energy consumption are development indicators. Countries releasing 15–20 tonnes of carbon per capita per year fall in the high emission category, while those with 10–12 tonnes on average are moderate emitters and those with emissions lower than 4 tonnes are considered low emission and comprise mostly of developing countries [10].

Pakistan, being a predominantly rural society, relies on solid fuel for household energy for cooking and heating. In order to conserve heat, the houses are kept closed in the northern part of the country which may raise the IAP levels several-fold compared to the southern part of the country where the houses are mostly open type due to the more temperate climate. Thirty-eight percent of the households consist of single-rooms, and presumably have greater IAP concentrations due to presence of the kitchen within them [7].

In Pakistan, the majority of the households rely on biomass fuels, mainly due to the unavailability of better alternatives. This, coupled with the small size of houses and improperly ventilated kitchens, leads to high levels of smoke and pollutants, thus increasing exposure and its associated negative health effects [11]. Moreover, it has also been observed that some households burn materials like polythene bags and plastic bottles along wood fuel to compensate for the low amount of wood available. This practice may result in increased amounts and types of pollutants being released. However, to our knowledge no studies have been done so far to document this practice or to quantify its ill effects. According to WHO estimates, the total number of deaths in Pakistan attributed to solid fuels is 70,700, while the percentage of national burden of disease attributed to solid fuel use is 4.6%, as compared to less than 1% seen in the developed world [9]. These figures put Pakistan among the 21 worst affected countries by IAP along with Afghanistan, Niger, Ethiopia and Rwanda, among others, according to WHO estimates [12].

Literature regarding IAP estimates is sparse. Small scale studies have been done in Pakistan. A study conducted in the city of Lahore compared indoor and outdoor levels of and variations in particulate matter (PM). High indoor levels of PM were observed during cooking, cleaning and smoking. PM levels in kitchen during cooking were between 4,000 to 8,555 μg/m^3^, while the typical range was from 200 to 5,000 μg/m^3^ among households using solid fuels [13]. Similarly, a study attempted to measure variations in indoor/outdoor concentrations of PM comparing rural and urban sites in Punjab province using spectrophotometers. The indoor as well as outdoor levels of PM in both sites were found to be much higher than the international standards, however, the kitchens using biomass fuels showed the highest indoor levels of PM, with indoor/outdoor ratios for PM_10_ and PM_2.5_ of 3.80 and 4.36 times, respectively [14].

In addition, another study done in a semi rural setting attempted to measure carbon monoxide levels using an electrochemical monitor. Measurements were made for eight continuous hours during the day. The levels of CO were found to be significantly higher among users of wood (29.4 ppm) compared to those using gas (7.5 ppm) [15].

Another study conducted in 2008 by Khudadad and Shah on a sample of 68 households in a village of northern Pakistan, showed a mean concentration of PM_2.5_ in the indoor environment of 7,380 μg/m^3^ while the outdoor environment showed a mean level of 80 μg/m^3^ [16]. The indoor readings given in these studies are far greater than those observed in developed regions. Although the levels of IAP generated through these studies cannot be generalized for all biomass users in Pakistan, the extreme levels of exposure reported are alarming, at least for a section of a population. According to the European EXPOLIS study the mean levels range from 9.5 μg/m3 to 35.6 μg/m^3^ in different European cities [17].

### 3.2. Health impacts of indoor air pollution

There is dearth of health impact studies in the developing world regarding the quality of indoor air and the risk it poses to health [5]. Despite the burden of ill health contributed, it has not been given its due importance in Pakistan. The studies conducted in Pakistan are mainly related to respiratory diseases and symptoms and pregnancy outcomes.

#### 3.2.1. Respiratory diseases and symptoms

Biomass fuel use has an association with acute upper and lower respiratory infections, otitis media, chronic bronchitis, chronic obstructive pulmonary disease, lung cancer, asthma and pulmonary tuberculosis. These are caused by respiratory irritants such as nitrogen, sulphur and carbon oxides, polycyclic aromatic compounds and carcinogens such as benzopyrene, among others.

A study to compare villages using biomass fuels with villages using gas in suburban areas of Peshawar city found strong association between chronic bronchitis and use of wood, dung cake, rice straws and maize (*kai)* grass. The highest association was seen with rice straws (OR 3.32) followed by wood (OR 2.38) [18]. In Pakistan ARI is the leading cause of mortality among children under 5 years. This age group spends more time indoors, so exposure to cooking smoke makes them twice (OR 2.0) as likely to develop ARI as compared to other children [19].

Considering that 86% of the households use biomass in Pakistan, the loss of disability adjusted life years (DALYs) due to IAP would be enormous. Women are primarily involved in biomass combustion for cooking and, therefore, inhale poisonous chemicals. Chronic obstructive pulmonary disease (COPD) among females and lower respiratory infections among children contributes to 45% and 41% of DALYs due to indoor smoke from biomass fuels, respectively. Also an increased risk of cancers (tracheal/bronchial/lung) among females has been noted in this region compared to males [20].

Health effects of IAP depend on the concentration and mix of pollutants and the level of exposure to the pollutants. World Bank estimated that IAP cause over 280,000 deaths a year and around 40 million cases of acute respiratory illnesses in Pakistan [20]. Chronic bronchitis is responsible for 22,000 DALYs lost per 10,000 cases, hospital admissions due to respiratory conditions contribute to 160 DALYs lost per 10,000 cases and lower respiratory illness in children lead to 65 DALYs lost per 10,000 cases, while morbidity attributed to ARI among females over 30 years is responsible for 8% of total DALYs, while that caused by COPD is around 5% of the total [20]. A meta-analysis of studies based on the burden and impact of IAP in developing countries has yielded a relative risk of 2.3 for developing ARI among children under 5 years of age among those exposed to IAP with a range of 1.9 to 2.7. It is estimated that the annual incidence of ARI per child under 5 years of age is 4.1 and 2 week prevalence is 24%. Some estimates imply that a 10 percentage point decrease in the share of energy consumption due to biomass fuels for a country lowers its child mortality rate by roughly 7 deaths per 1,000 live births [20], while that for COPD in women over 32 years of age showed a relative risk of 3.2, with a range of 2.3 to 4.8. This clearly indicates the vulnerability of those exposed to the damaging effects of IAP. The extent of damage, however, varies depending upon the level of pollution in the indoor air and amount of time individuals are exposed to it [21].

Besides biomass, tobacco related health and economic hazards also contribute to IAP in Pakistan. People smoke in closed and confined spaces like inside homes, office rooms and buses [22]. People also smoke at public places because of ineffective legislation to control smoking [20]. The contribution of smoking to IAP is assumed to be significant, especially in houses where biomass is not being used, however it requires an estimation of the behaviors as to how much proportion of individual also smoke indoor inside the house.

New cases of ARI and COPD morbidity and mortality occurring each year that can be attributed to use of solid fuels can be calculated by the following equation:

(1) $$\left[{\text{D}}_{\text{i}}=\text{PAR}\;*\;{\text{D}}_{\text{i}}^{\text{B}}\right]$$

where D_i_^B^ is baseline cases of morbidity and mortality, while PAR is calculated by:

(2) [PAR =PP*(OR−1)/(PP*(OR−1)+1]

where PP is the percentage of population exposed to solid fuels and OR is the odds ratio. Based on the above estimation technique ARI morbidity among children and females and ARI child mortality from IAP represents about 38–53% of total ARI in Pakistan [19].

#### 3.2.2. Effects on pregnancy outcomes

Low and middle income countries have a considerable burden of still births and neonatal deaths. These are attributed to a variety of factors that adversely influence the health of the mother and the unborn child such as lack of antenatal care, malnutrition among females and tobacco smoke. It has also been documented that exposure to smoke during cooking from biomass fuels that contains carbon monoxide and particulate matter may lead to fetal hypoxia or oxidative stress which can result in impaired fetal growth. Studies conducted in Pakistan as well as other neighboring countries have shown a significant increase in the risk of stillbirths among those exposed to smoke from inefficient biomass fuels. One study has shown a nearly two-fold increase in risk of stillbirth among women who are utilizing biomass fuels during pregnancy [23]. The association with still births is further reenforced by a multi country systematic review utilizing data from Pakistan among other developing countries. It yields an odds ratio of 1.51, thereby indicating 51% increase in the risk of stillbirths as a result of IAP. [24] One possible explanation for this association is the high levels of CO in the maternal and fetal circulation, reducing the oxygen carrying capacity of blood and thus producing fetal hypoxia, an important contributor of IUGR and ultimately still birth.

Biomass smoke has also been found to be associated with low birth weight (LBW) deliveries. It is estimated that in Pakistan around 21% of infants are born with a birth weight of less than 2,500 g, thus qualifying them as LBW. Evidence regarding association of LBW with use of biomass fuel use and IAP is still insufficient to a large extent and very few quantitative studies have been done so far in Pakistan. One study was conducted on 1,404 pregnant females recruited from surrounding areas of Karachi that included both urban and rural locations using mostly wood fuel. Greater occurrence of miscarriages among wood fuel users were reported compared to natural gas users. In addition, it was found that the odds of having LBW were 1.7 times among biomass users compared to natural gas users. [25] In Pakistan, poor nutrition and reproductive outcomes in combination with exposure to biomass fuel smoke further aggravates and compounds the effect [15]

A prospective cross sectional survey done in low and middle income countries that included data of over 800 pregnant females from Pakistan as well, attempted to gather information regarding their exposure to IAP. It concluded that majority of the pregnant females *i.e.* 87% were exposed to some form of solid fuel, mostly crop residue and wood, during their pregnancy. In addition, it was seen that households using the poorest quality of fuel were also more likely to allow indoor smoking, thus further deteriorating the quality of indoor air. [26]

### 3.3. Cost of health impacts

IAP in Pakistan leads to a total annual cost of around 55–70 billion rupees. This cost is related to expenses incurred to provide medical treatment for sick, time lost and cost attributed to mortality for adults and children using Schulman methodology [27]. IAP affects the health of the poor and underprivileged population [28]. It was estimated that annual cost of environmental and natural resource damage is about 365 billion rupees per year which amounts to 6% of GDP in Pakistan. Among this the third highest cost was contributed by IAP which was responsible for loss of 67 billion rupees or 1% of GDP. Urban air pollution contributes a further 65 billion [28]. IAP clearly represents a significant economic burden and remains an issue that warrants considerably better policy and analytical attention than is currently given to it.

### 3.4. Mitigation efforts and capacities

According to an earlier analysis done on the situation of IAP, it was concluded that IAP is still not considered a well recognized health hazard among the general population as well as the scientific community. Very few large scale studies have been carried out to quantify the effects of IAP on healthy and to test efficacy of the available intervention options [29].

#### 

##### Public awareness and policy formulation and standards

Addressing IAP effectively will require multiple steps to be taken simultaneously including raising the awareness among government decision makers, non-governmental organizations (NGOs), and the population in general. Involvement of authorities for development of policies has the potential to bring about substantial changes. Substitution and provision of alternate fuels is not possible in near term in Pakistan. Aversion and cost-effective risk reduction approaches requires development of policies and standards for households.

##### Improved cooking stoves

A fuel-efficient cooking technology project, funded by GTZ (Germany) and providing improved stoves, was launched successfully and implemented throughout Pakistan. However, no further expansion was done later on [20]. PCRET (Pakistan Council for Renewable Energy Technologies) installed 60,000 improved cooking stoves all over the country that conserve energy and their efficiency has shown to be between 12–28% [30]. The stove efficiency reduces the amount of firewood needed and thus exposure; and it also saves time spent on collecting the fuel. They are affordable and easy to use. They offer a practical solution for the problem as against use of alternate fuels which prove costly for the poverty stricken households. Through this effort, however, the hazard is partially but not completely abated.

A pilot study conducted in interior Sindh, a province in Pakistan, has shown that women felt more favorably towards improved stoves (made of clay with chimneys) in terms of the amount of smoke that is released. In addition, the mean levels of carbon monoxide in kitchens using improved stoves were lower than those observed in traditional stoves (three-stone stoves) using kitchen [31]. A similar initiative was taken by an NGO by the name of Escorts Foundation. With the help of UNDP funding, they introduced fuel-efficient stoves in 24 villages in Changa Manga, a forest area of Punjab. These interventions were made to be cost-effective and sustainable by using recyclable material for making chimneys. This initiative has reduced the amount of wood fuel used daily by half, thus saving both, time and energy [31].

##### Biomass conversion systems

Renewable energy resources are energy resources that are replaced rapidly by natural processes such as sunlight, hydropower *etc.* Conversion of dung into biogas is common in many South Asian countries like India, China and Nepal. Government of Pakistan started a comprehensive biogas scheme in 1974 and commissioned 4,550 biogas units by 1990 throughout the country. The units were designed to provide 3,000 to 5,000 cubic feet of biogas per day for cooking and lighting purposes. Unfortunately, after the withdrawal of the government financial support, the project did not progress any further [20]. Such projects need to be activated looking at the constraints and lessons learned. The longer term solution for increasing demand from the population lies in switching to renewable energy technologies. For example, India is claiming to draw at least 15% of their total energy from sunlight by 2020. Even if such targets are underachieved it would still provide opportunity for better investment in future.

##### Enhancing access to modern fuels

Facilitating the uptake of natural gas among urban households through gas pipeline expansion is an option, but the expansion of gas to rural areas should be looked at carefully that whether poor would be able to pay for the connection charges which would be initially required [32]. Switching to cleaner fuels will have positive impact on health, but poor households may face difficulties in paying for the installation and monthly expenses. Also, these resources are not unlimited and may not be enough for whole population.

##### Housing interventions

Housing intervention is another possibility to reduce IAP, but very little attention has been paid in this regard so far in Pakistan. Practically it is difficult for households to modify building structures or rebuild the infrastructure and for many it is therefore not feasible. However, it should be considered for new houses which are going to be built in future. Housing interventions have several additional favorable health effects in addition to abatement of IAP. Small scale interventions have been done in northern Pakistan by Building and Construction Improvement program with favorable impact [31].

##### Organizational capabilities

There is serious lack of human and organizational capabilities in monitoring and developing interventions for indoor air quality. Several governmental organizations and NGOs are workingin the area however no collective effort is being seen at the national level.

## 4. Conclusion and Recommendations

The paper attempts to present assimilated available information on IAP in Pakistan. Environmental health is a relatively new area of research therefore information on environmental statistics in general and on IAP in particular is rudimentary in Pakistan. Only sporadic efforts are being carried out to quantify the health effects and to determine associations with various health conditions, therefore there was great need to document such activities to determine the way forward.

The published literature and reports either do not provide direct evidence from the data or it does not count IAP as an environmental health issue for Pakistan. Most of the documents available are focused on ecological environmental health and the issue of human health has been neglected. The available information had two main purposes: to show progress *viz.* forestry and woodfuel conservation. In addition, the information gathered is sporadic and few well-conducted studies are available.

Nonetheless, most of the households are utilizing biomass as main source of energy in Pakistan. This is exposing a large section of population to indoor hazards. It is also depleting forests (deforestation) at an alarming rate. The women and children, who live mostly indoor, due to less outdoor work and less school enrollment ratios, are primarily getting exposed to IAP in Pakistan. Poverty is intricately linked as it is forcing households to use less preferred smoke-generating lower quality wood fuels, dung and agriculture residues.

The situation warrants the formulation of policy regarding IAP in Pakistan *viz*. recognizing it as a major hazard for the population, especially women and children; setting pragmatic and achievable standards regarding indoor air; directing attention of stakeholders towards developing interventions to decrease IAP in Pakistan. Development of national standards by Environmental Protection Agencies (EPA) regarding IAP would also help expedite the process. At the moment, there is no regulation which gives any guidelines regarding IAP levels in indoor settings in Pakistan.

The data is sparse regarding the health consequences due to IAP in Pakistan. Data and evidence gets accumulated and needed over time. There may be less need for such data, however, once the health impact of IAP is clearly established. The attributable risk associated with IAP is well-established through the global comparative risk assessment. In this respect, well-conducted studies of effective interventions for reducing IAP and fuel consumption and its beneficial impact are needed. These interventions should be practical, acceptable by the users, and required to be sustainable, cost-effective and fuel-efficient. Pilot studies of several interventions have been carried out at various places within Pakistan. These efforts need expedition and collaboration to enhance its impact and scope.

One of the available and practical options to improve the situation of indoor air includes development and adoption of fuel-efficient and smoke-free stoves for population at large scale. This will have ‘double impact’ of improving the environment and health, primarily of women and children, and saving energy. Deficiencies has been identified in implementation of such interventions done at smaller scale, therefore, these can be addressed in future.

Besides improved stoves, other technologies already developed by Pakistan Council for Renewable Energy Technology (PCRET) should be introduced in the market on a mass scale. There is potential of using biogas as rural energy throughout the country by network of community biogas plants. Alternate fuels such as expansion of gas supply to rural areas with subsidies may be an option in Pakistan, although warrant caution due to its limited availability and requirement for initial investment. Overall, a holistic IAP control measures such as interventions in areas such as petroleum sector, small business development, improved stoves programs, rural poverty alleviation strategies and health education is required.

## 5. Limitations of the Review

This review attempt to do a situational analysis of IAP and its health effects, based on review of documents, published and unpublished reports from various agencies and organizations working in Pakistan. The information and analysis provided in these documents on many points felt incomplete, therefore determining a trend of IAP and use of biomass overtime could not be determined. The reported studies are largely observational and very few measured exposure directly, relying instead on proxy indicators such as fuel type, stove type or time spent near the fire. The main purpose was to communicate essential facts and deficiencies in the available information and to advocate policy and intervention on IAP in Pakistan.
